# Supplementary material for: Synthesis and Characterization of Low-Cost Cresol-Based Benzoxazine Resins as Potential Binders in Abrasive Composites
Source: Materials (Basel). 2020 Jul 5;13(13):2995. doi: 10.3390/ma13132995 (PMC7372411; doi:10.3390/ma13132995)
Supplement: Supplementary file 1 [file materials-13-02995-s001.pdf]

materials

Supplementary Materials

Synthesis and Characterization of Low-Cost Cresol-Based Benzoxazine Resins as Potential Binders in Abrasive Composites

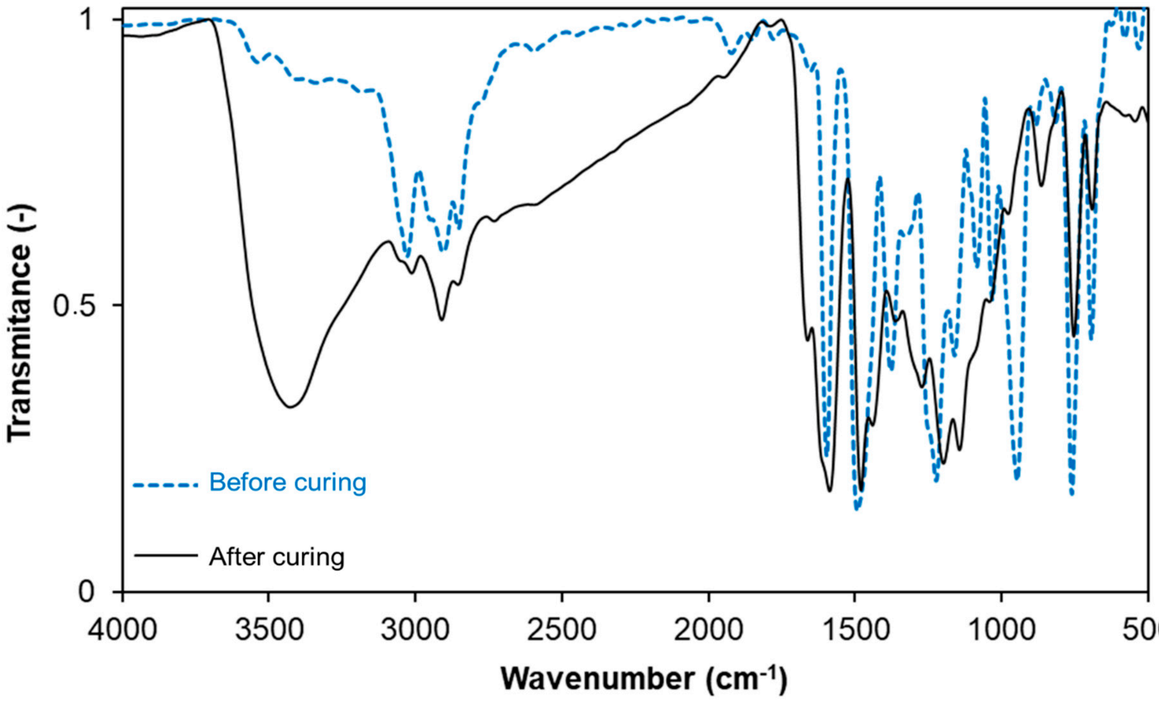

Figure S1. FTIR spectrum of *o*C-A benzoxazine.

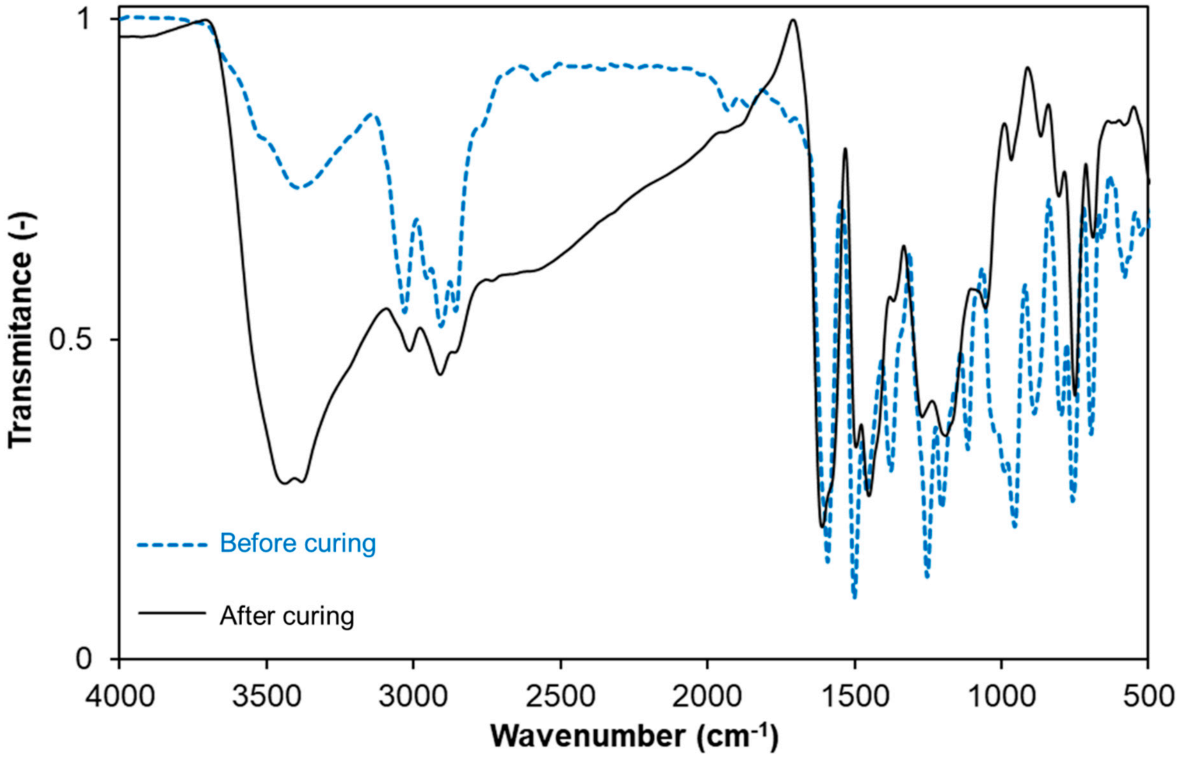

Figure S2. FTIR spectrum of *m*C-A benzoxazine.

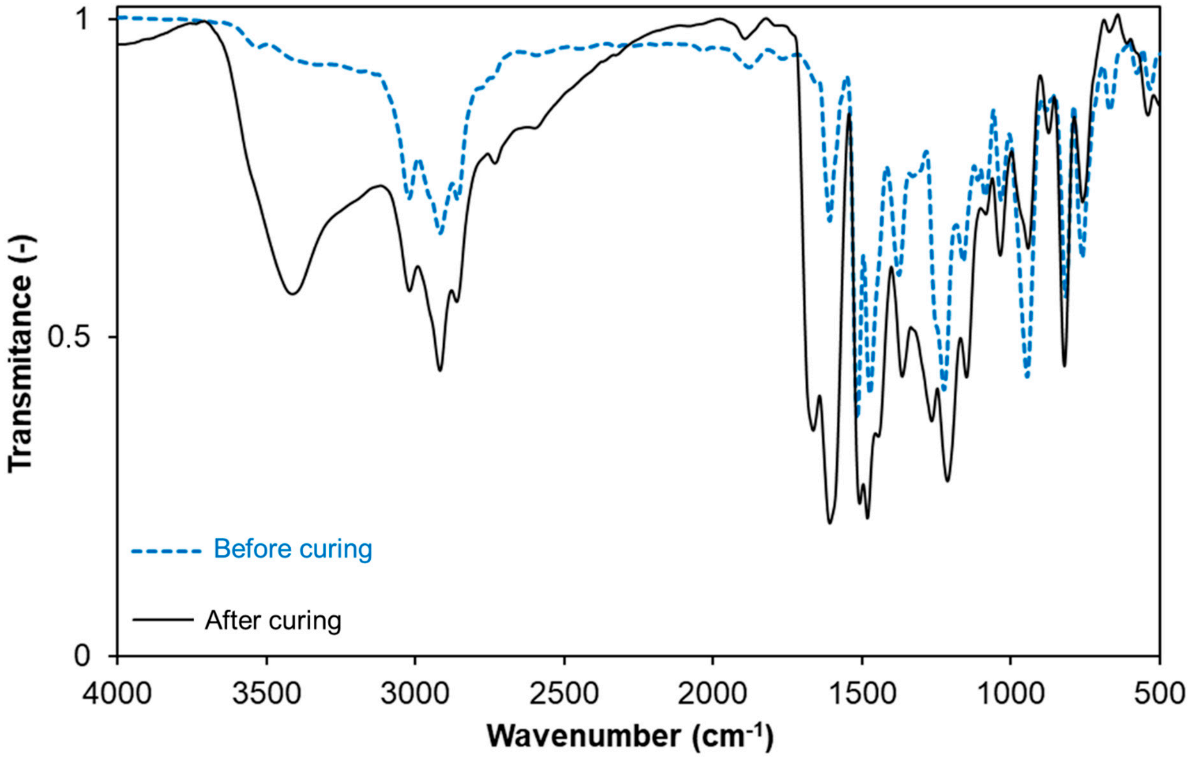

Figure S3. FTIR spectrum of *o*C-*p*T benzoxazine.

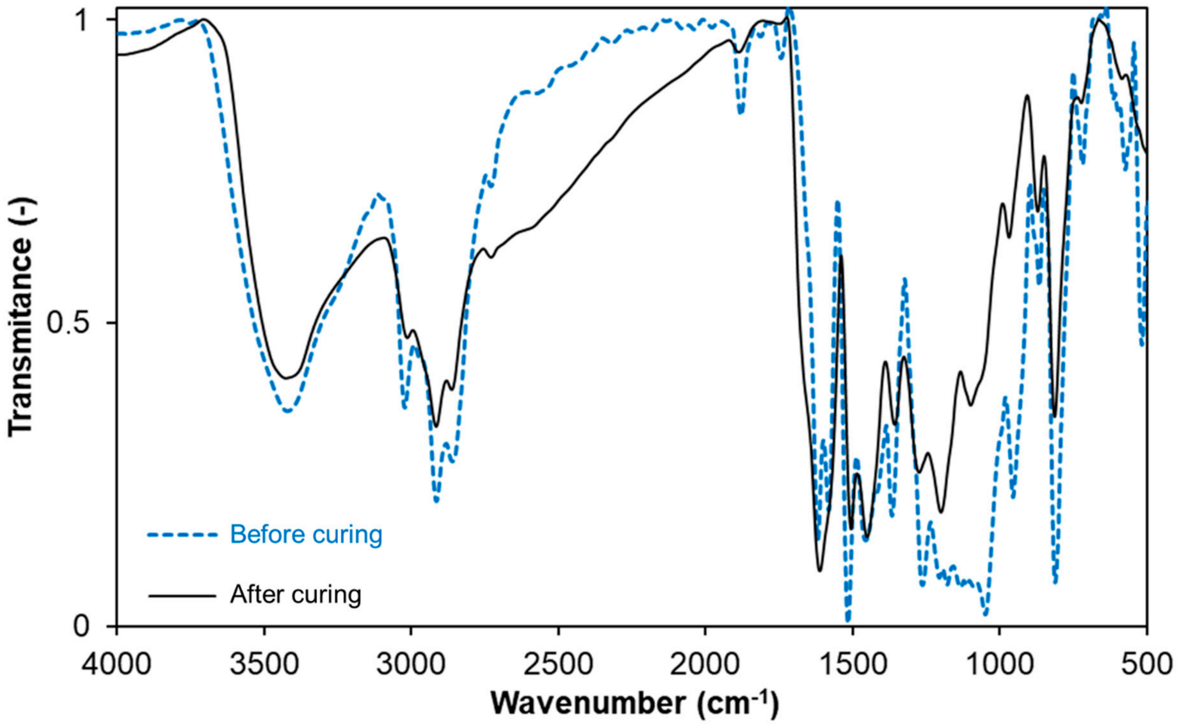

Figure S4. FTIR spectrum of *m*C-*p*T benzoxazine.

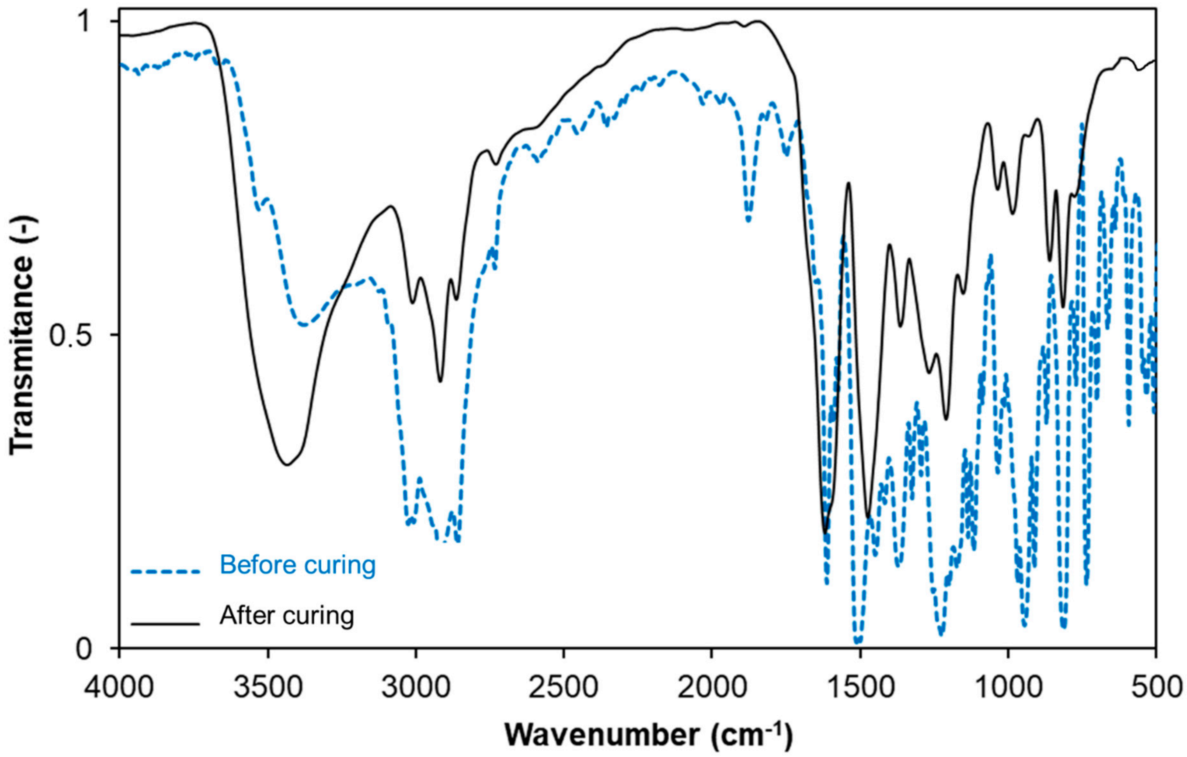

Figure S5. FTIR spectrum of *m*C-*p*T benzoxazine.

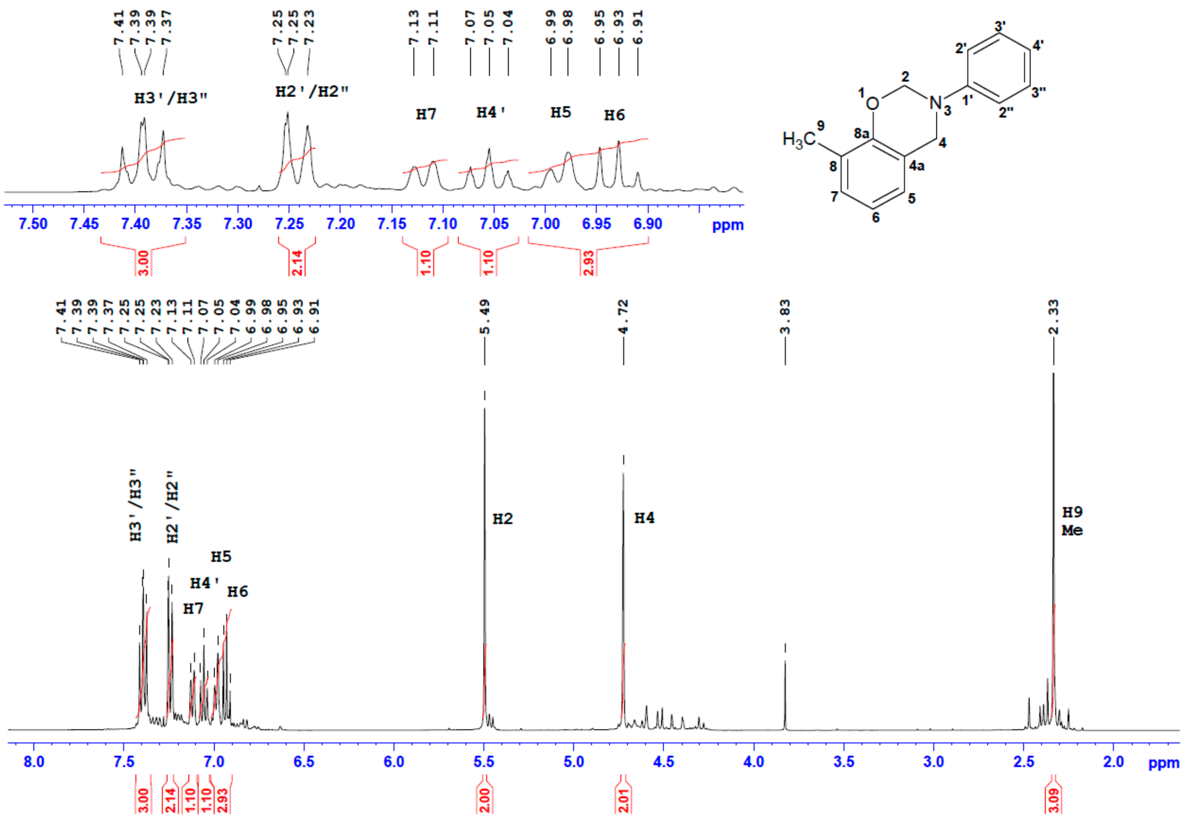

Figure S6. ^{1}H-NMR spectrum of *o*C-A benzoxazine.

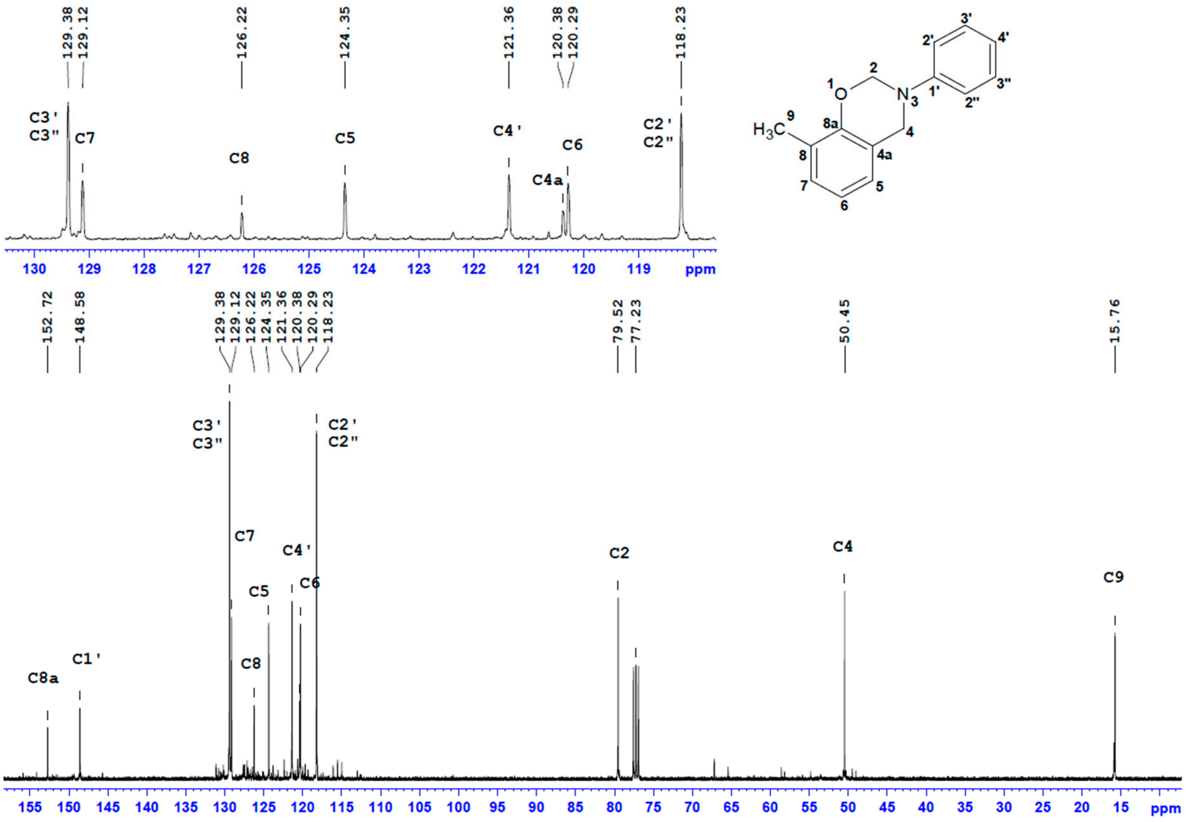

Figure S7. ^{13}C-NMR spectrum of *o*C-A benzoxazine.

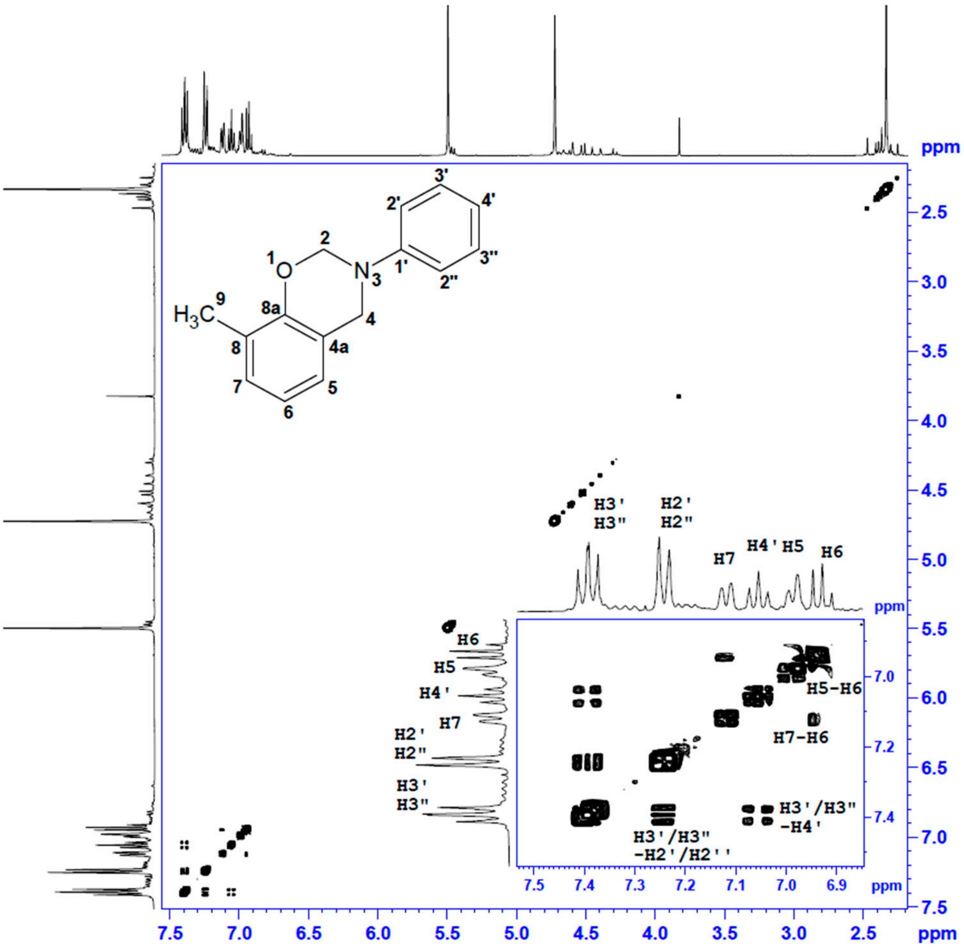

Figure S8. ^{1}H-^{1}H COSY spectrum of *o*C-A benzoxazine.

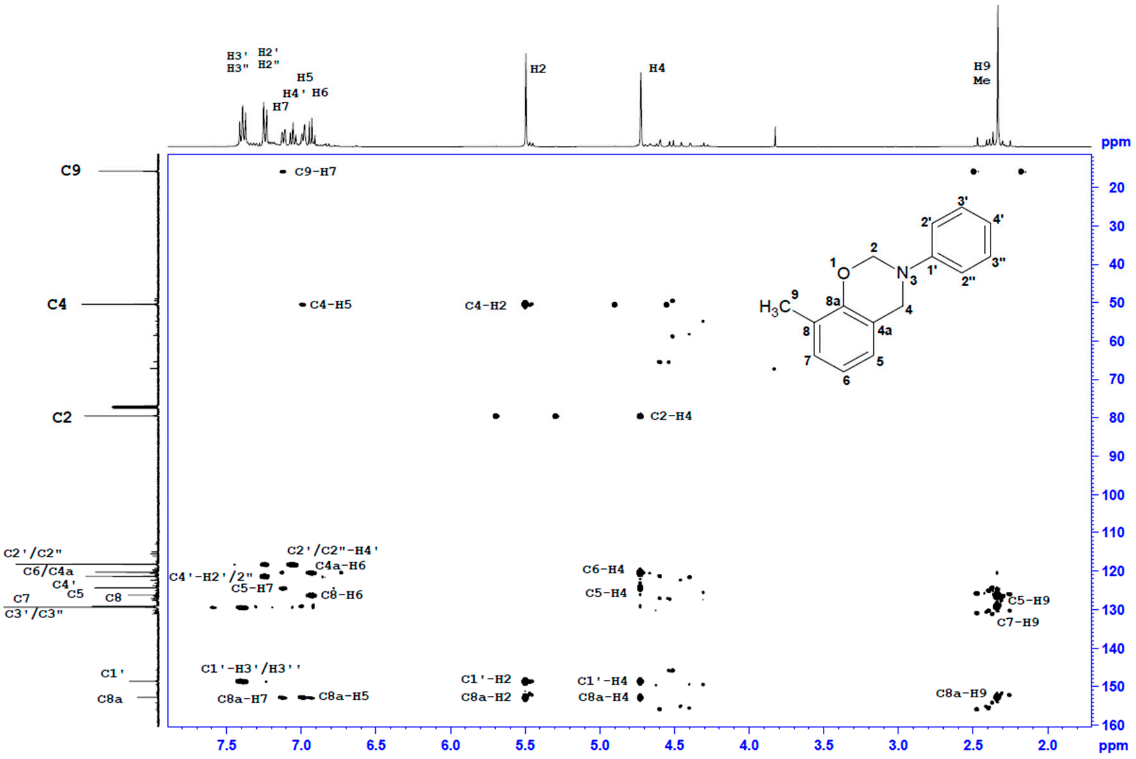

Figure S9. ^{1}H-^{13}C gHSQC spectrum of *o*C-A benzoxazine.

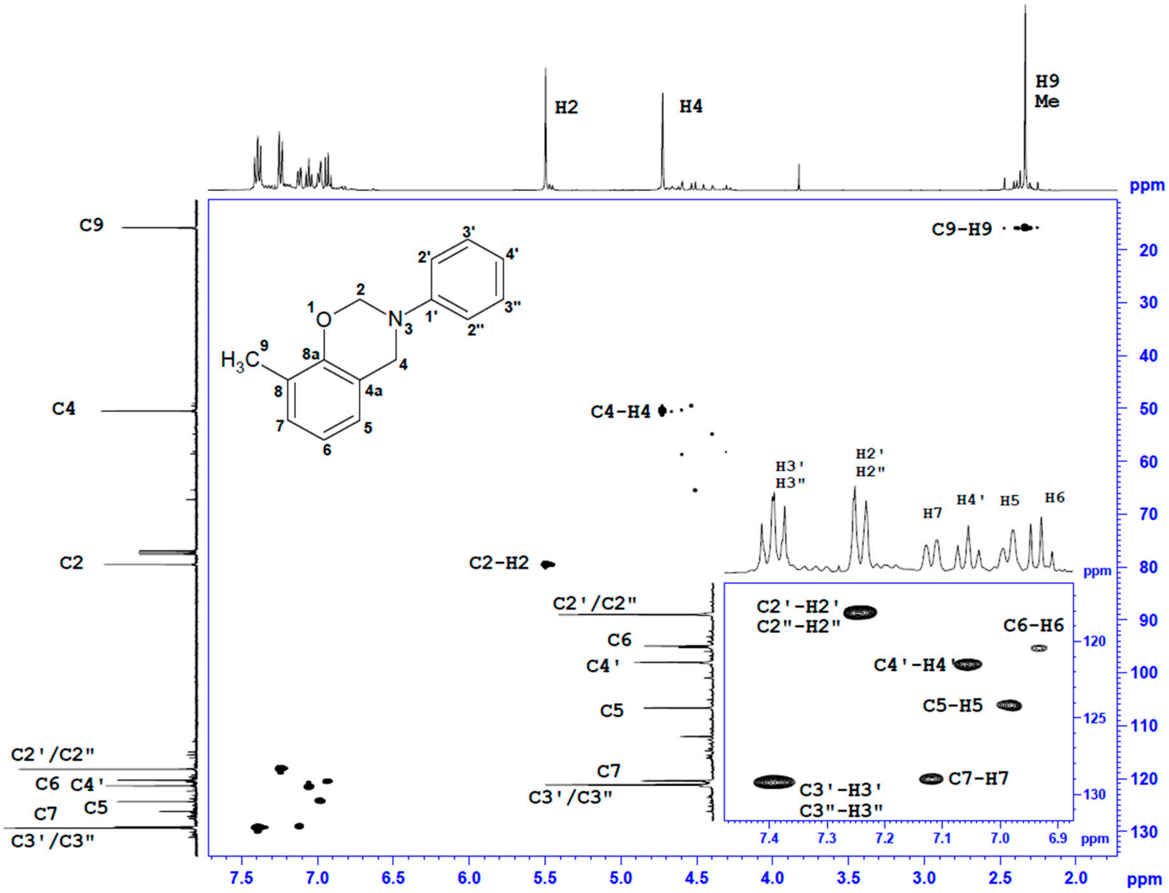

Figure S10. ^{1}H-^{13}C gHMBC spectrum of *o*C-A benzoxazine.

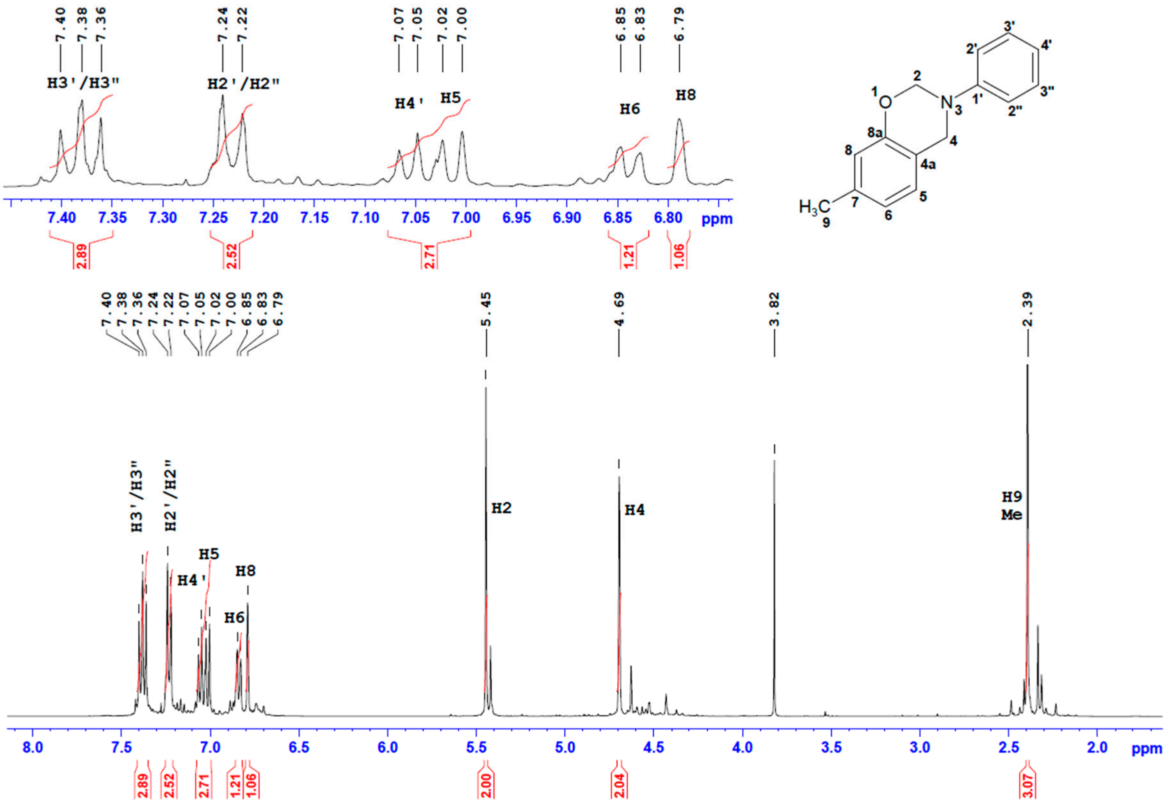

Figure S11. ^{1}H-NMR spectrum of *m*C-A benzoxazine.

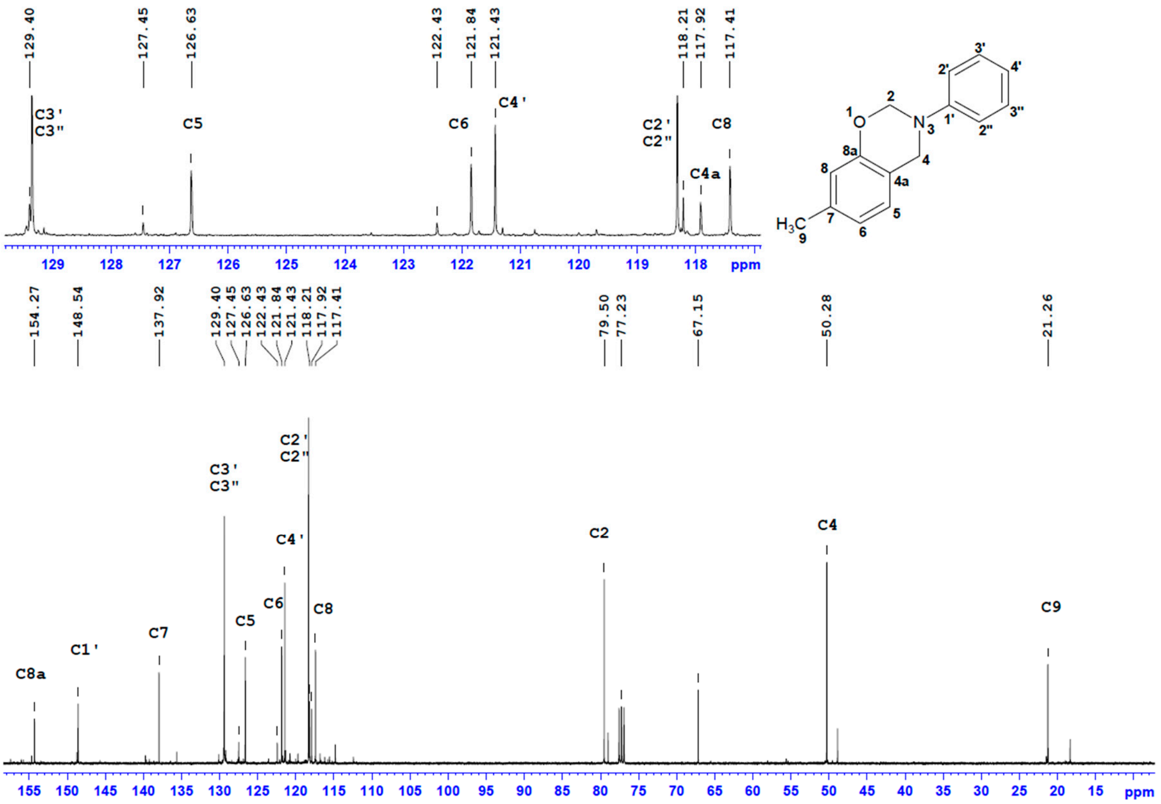

Figure S12. ^{13}C-NMR spectrum of *m*C-A benzoxazine.

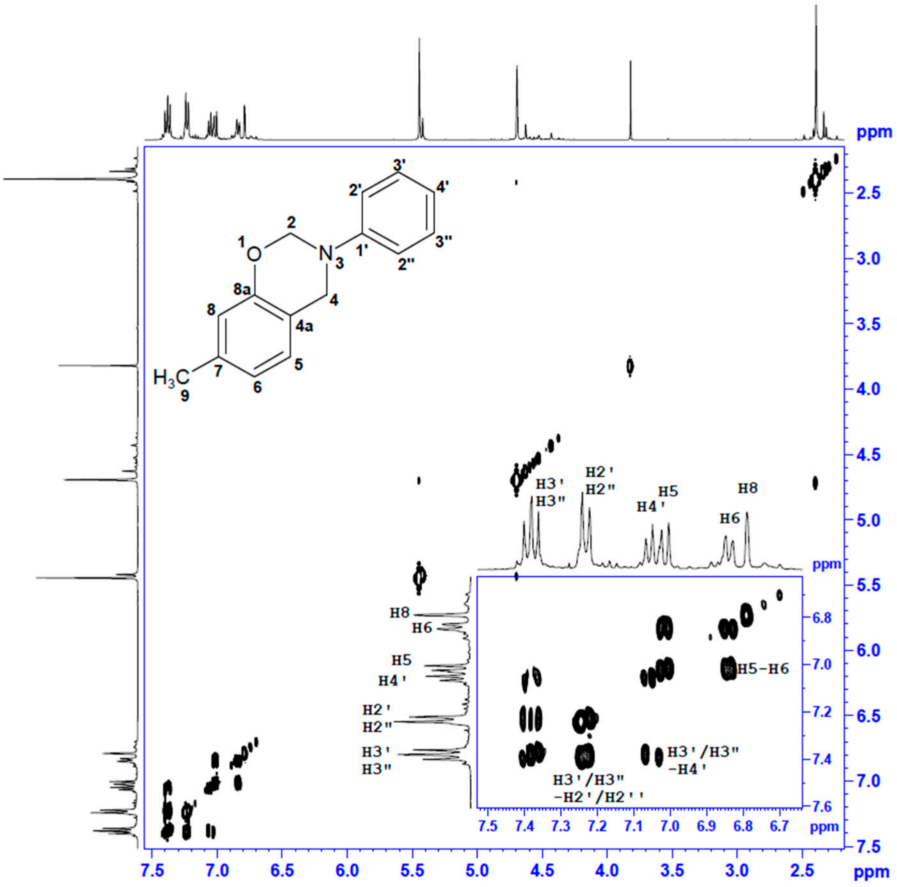

Figure S13. ^{1}H-^{1}H COSY spectrum of *m*C-A benzoxazine.

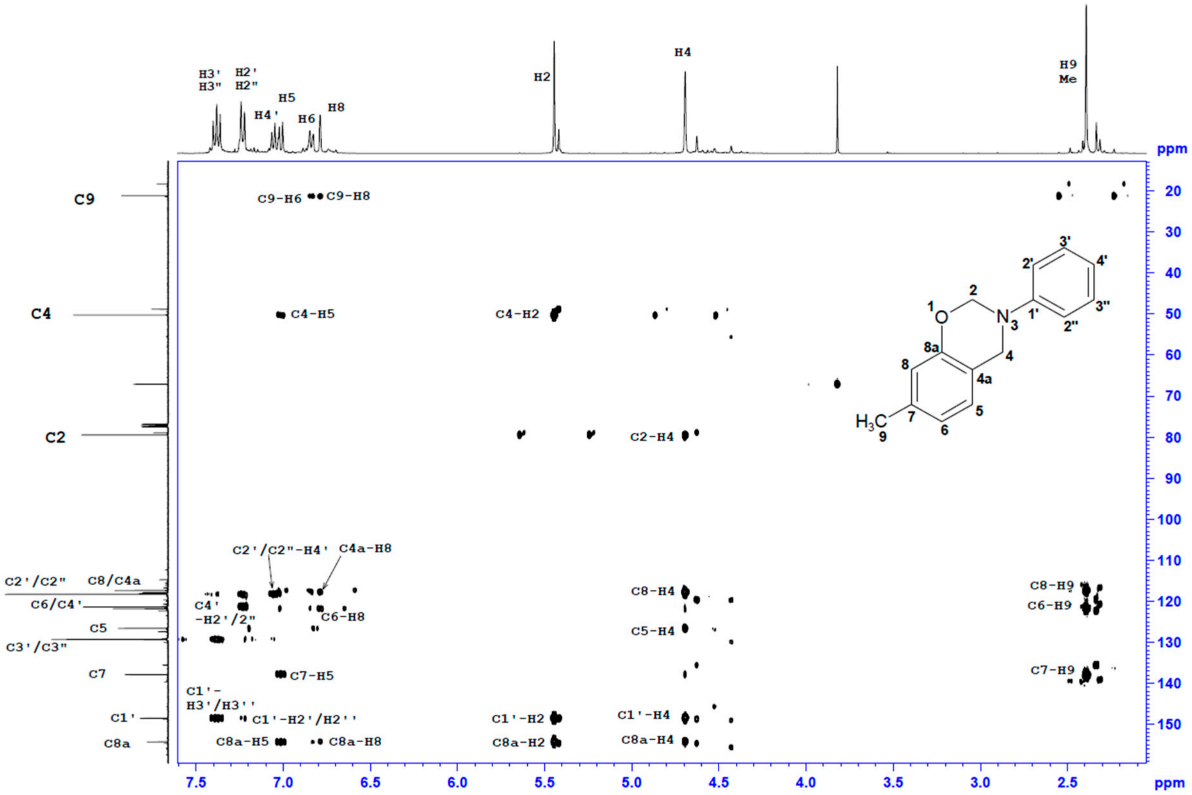

Figure S14. ^{1}H-^{13}C gHSQC spectrum of *m*C-A benzoxazine.

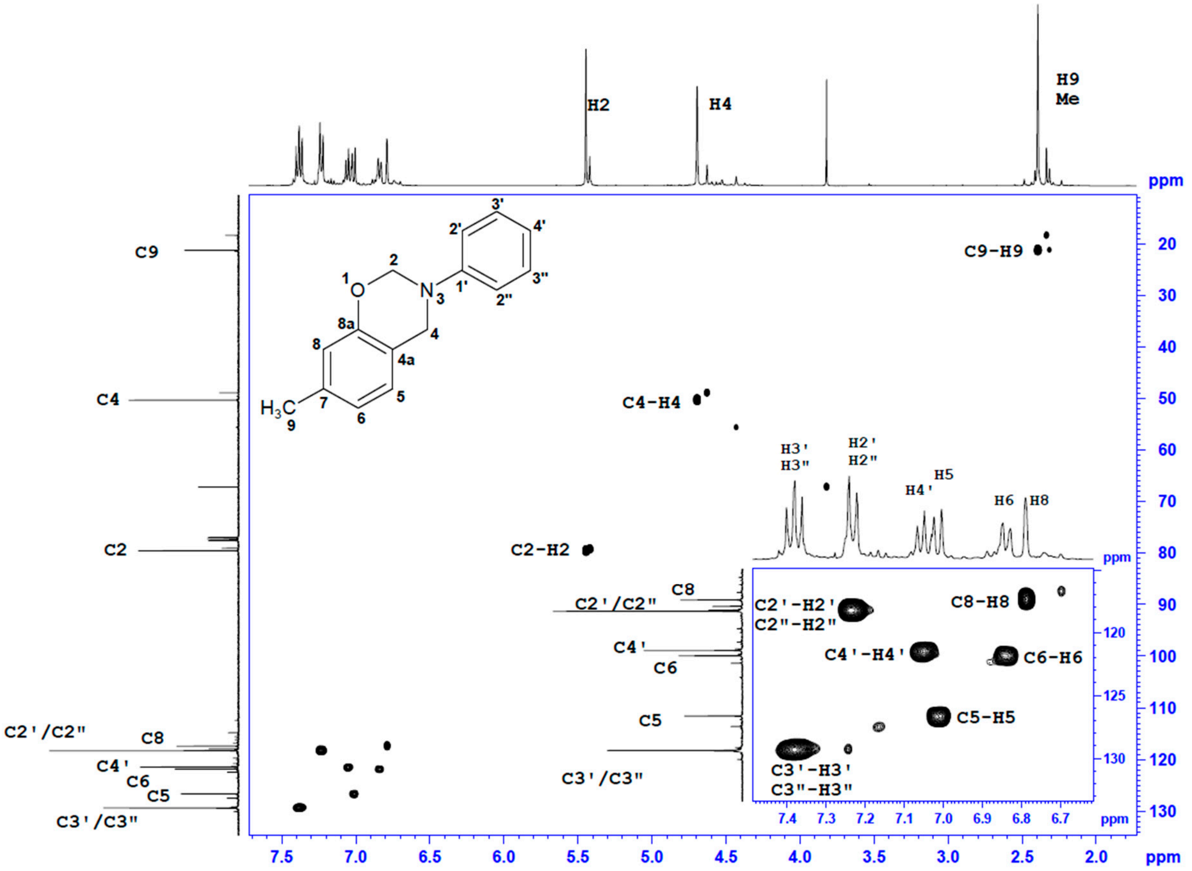

Figure S15. ^{1}H-^{13}C gHMBC spectrum of *m*C-A benzoxazine.

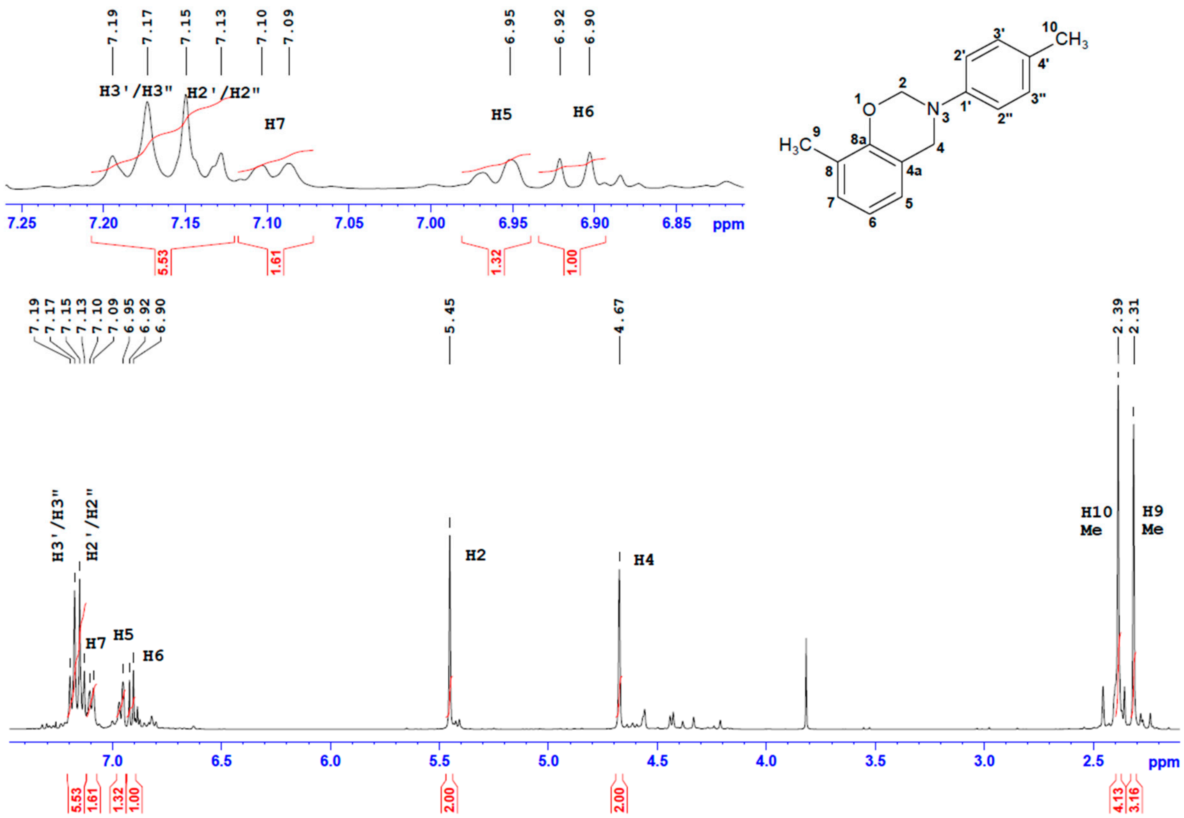

Figure S16. ^{1}H-NMR spectrum of *o*C-*p*T benzoxazine.

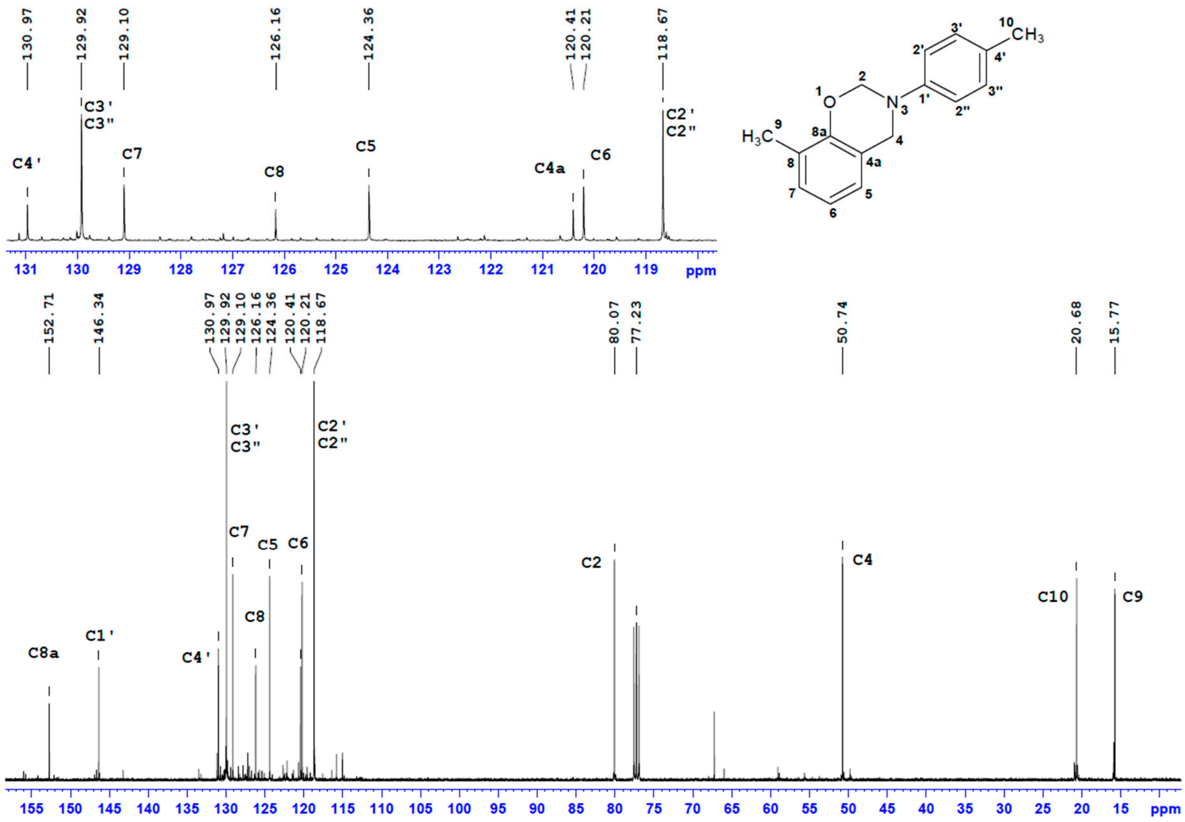

Figure S17. ^{13}C-NMR spectrum of *o*C-*p*T benzoxazine.

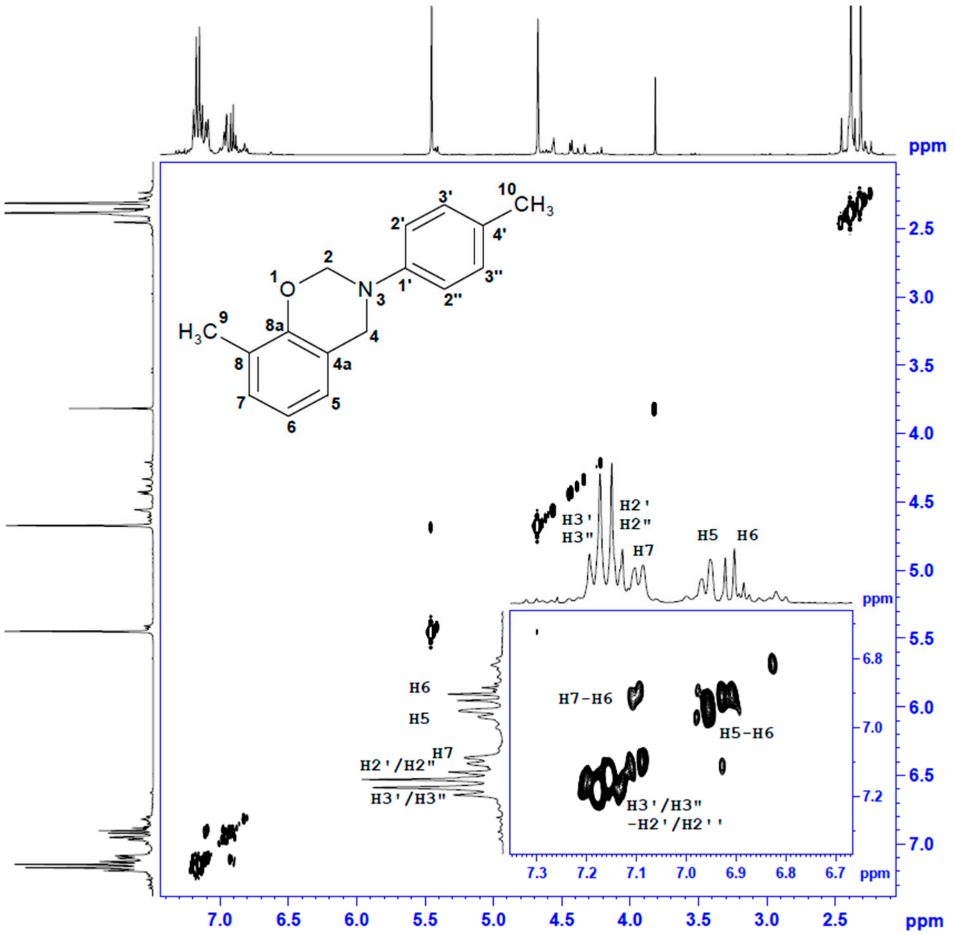

Figure S18. ^{1}H-^{1}H COSY spectrum of *o*C-*p*T benzoxazine.

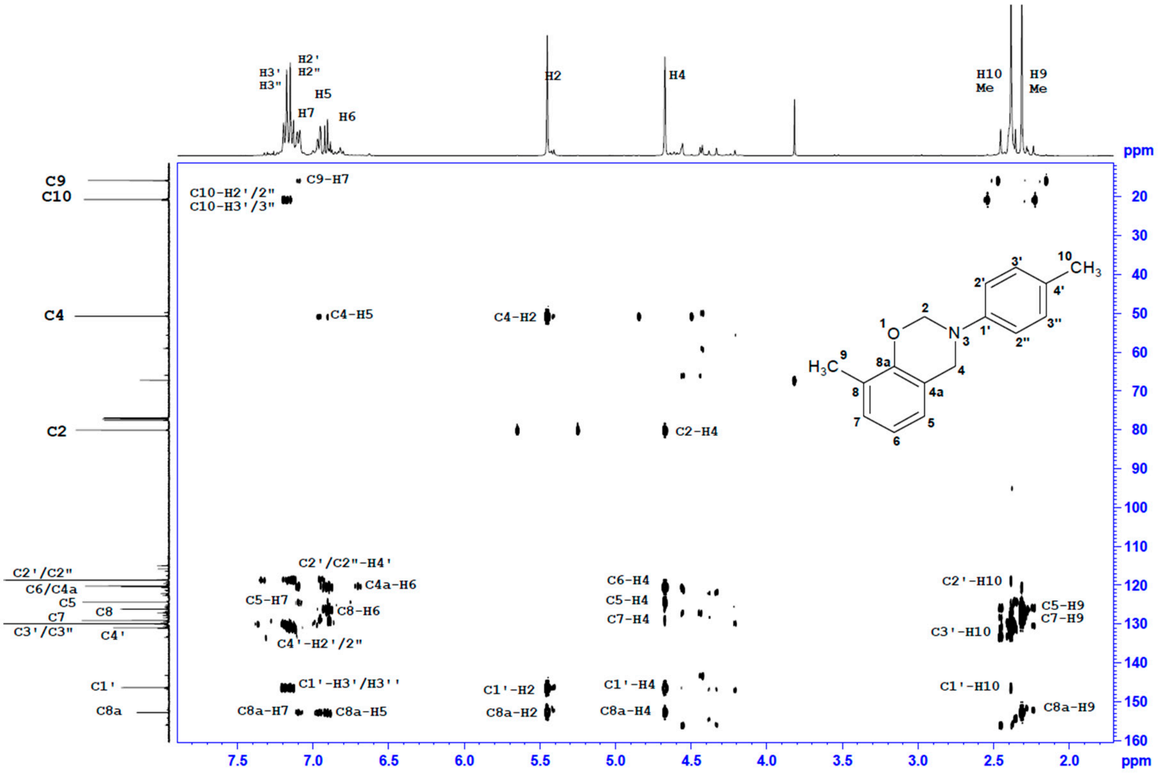

Figure S19. ^{1}H-^{13}C gHSQC spectrum of *o*C-*p*T benzoxazine.

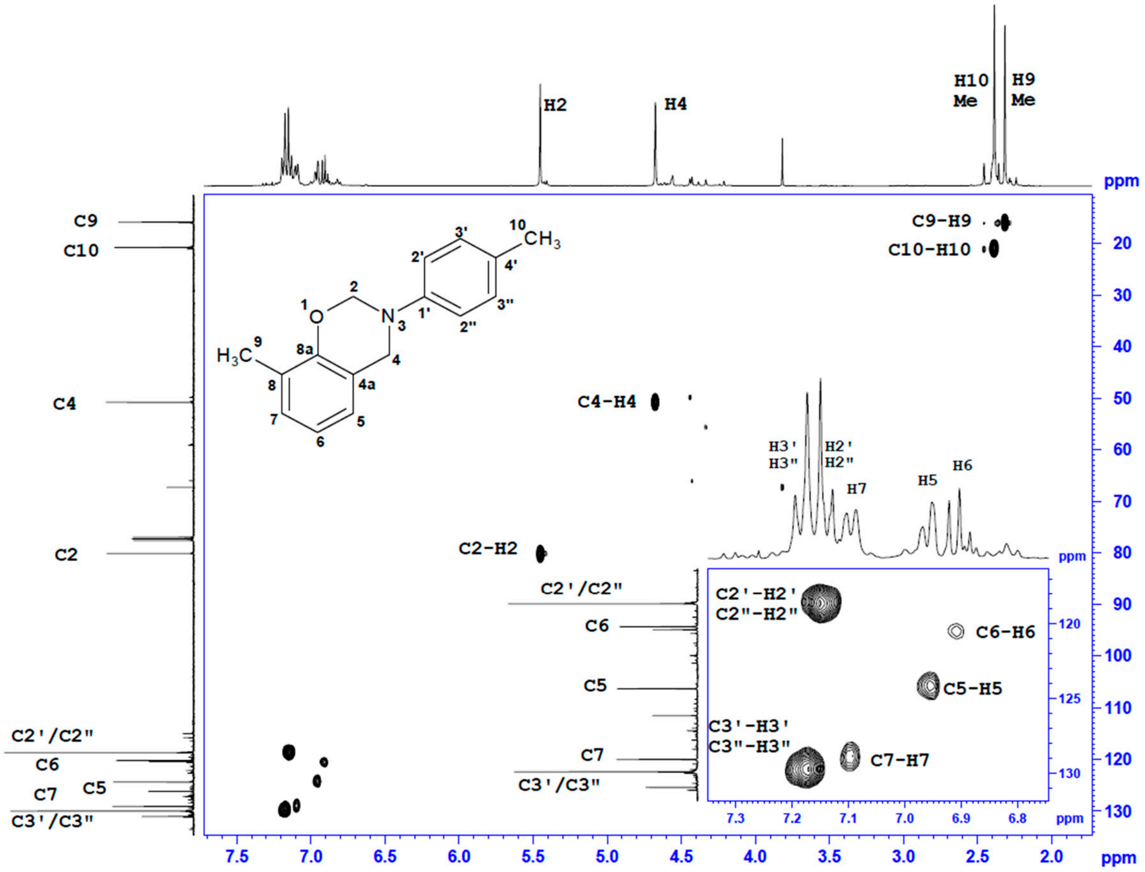

Figure S20. ^{1}H-^{13}C gHMBC spectrum of *o*C-*p*T benzoxazine.

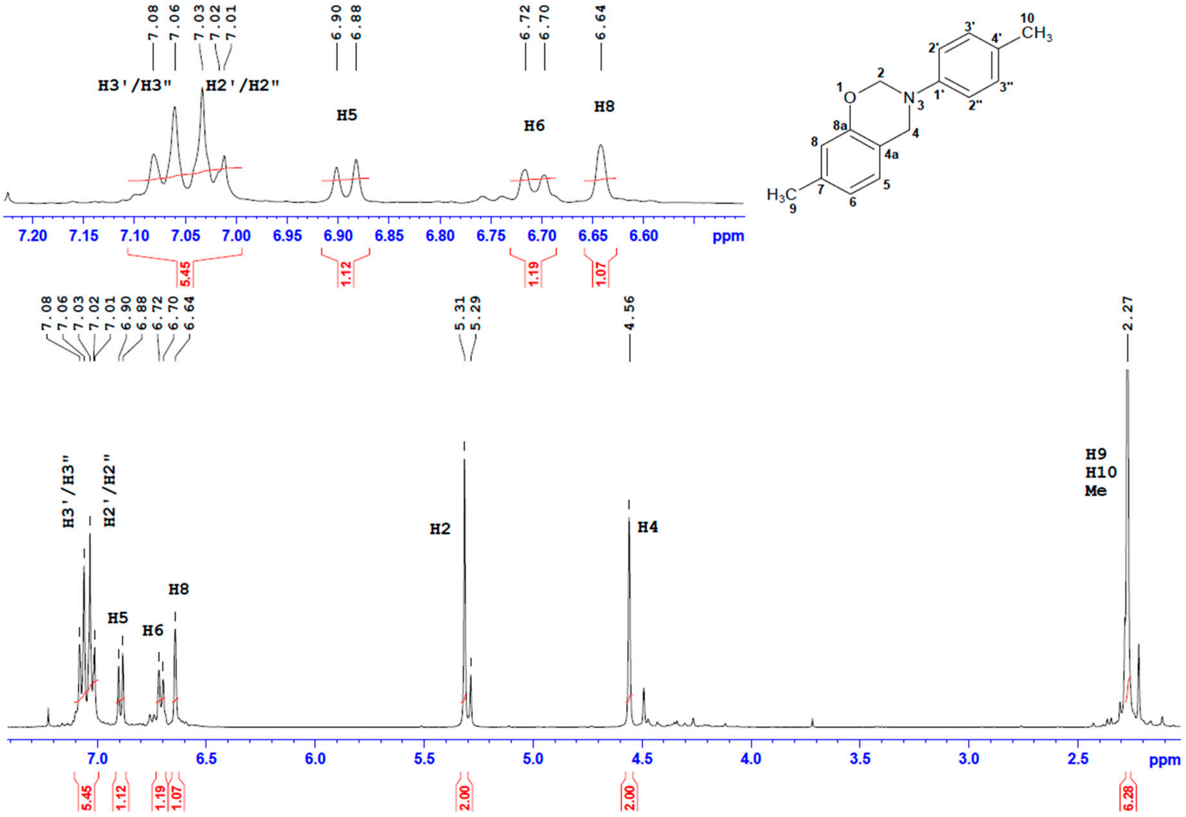

Figure S21. ^{1}H-NMR spectrum of *m*C-*p*T benzoxazine.

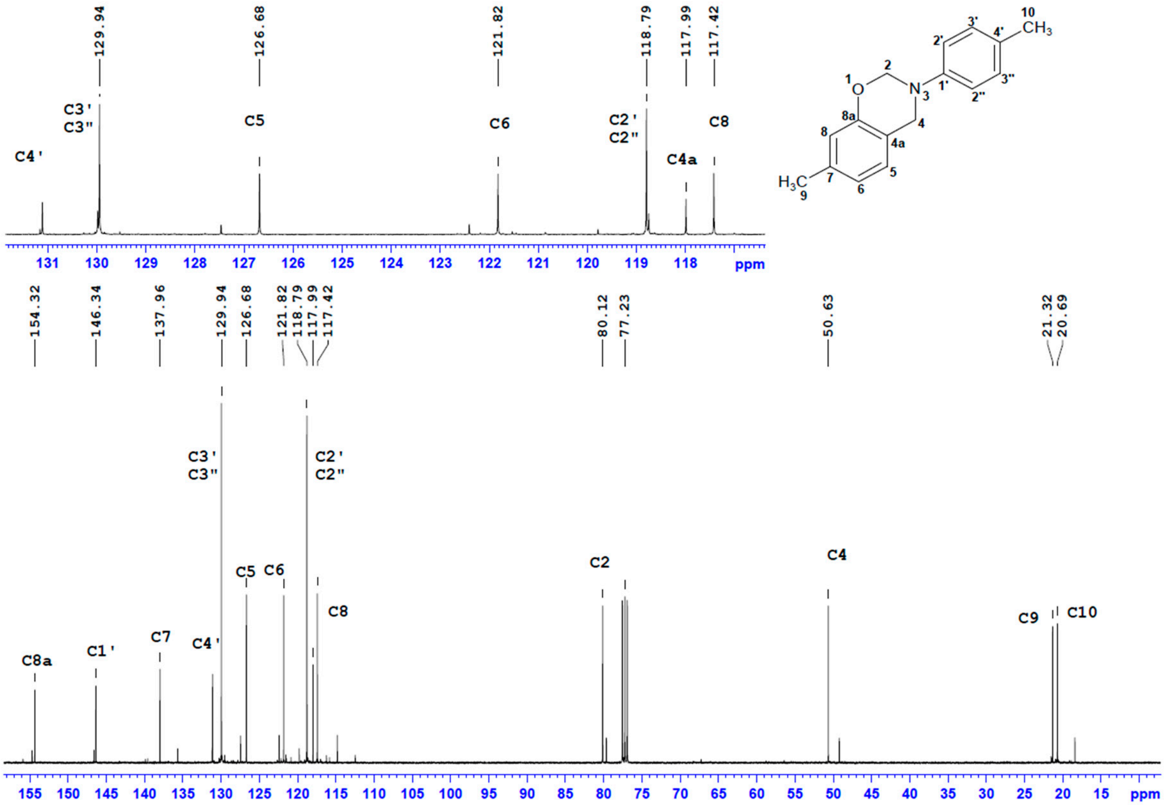

Figure S22. ^{13}C-NMR spectrum of *m*C-*p*T benzoxazine.

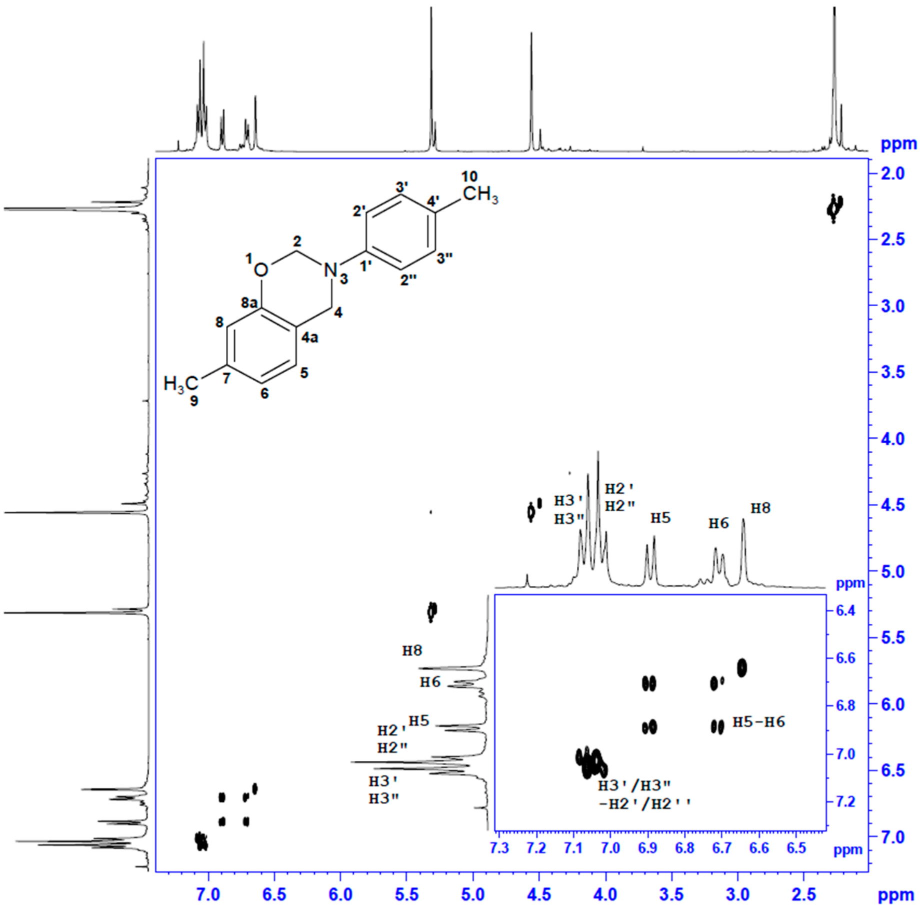

Figure S23. ^{1}H-^{1}H COSY spectrum of *m*C-*p*T benzoxazine.

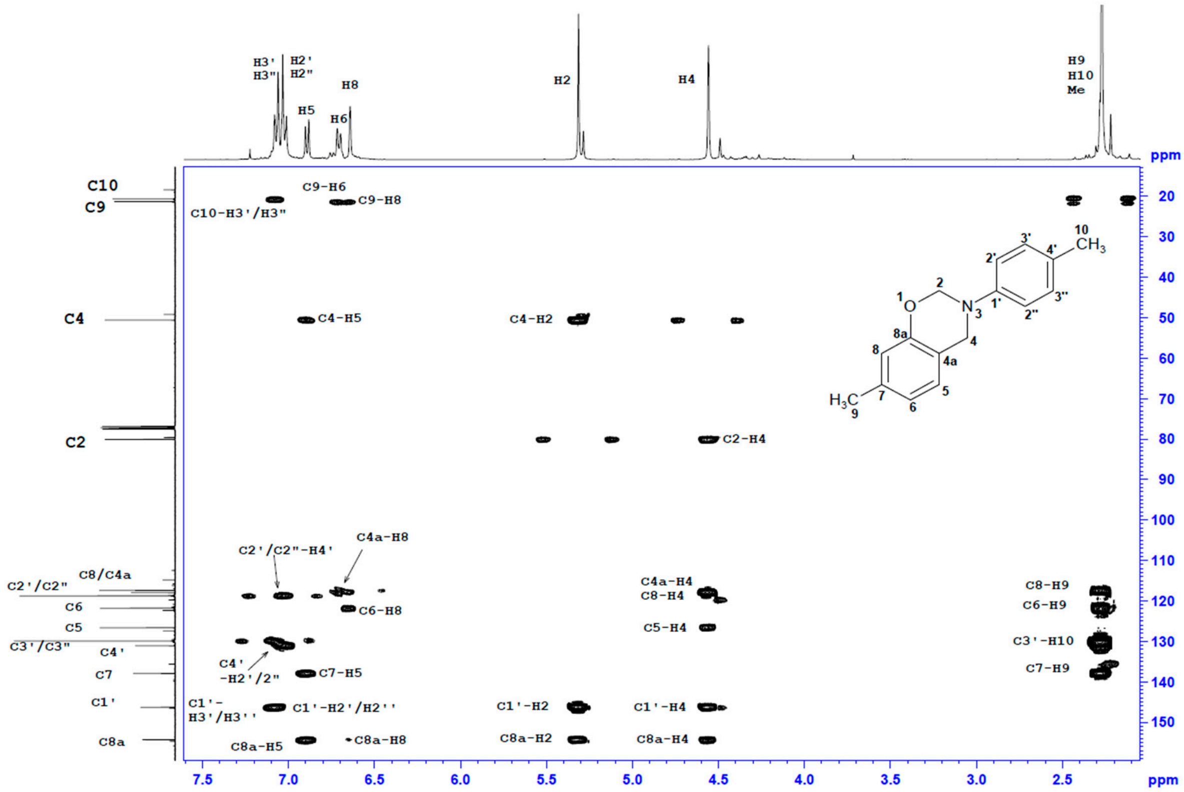

Figure S24. ^1H-^{13}C gHSQC spectrum of *m*C-*p*T benzoxazine.

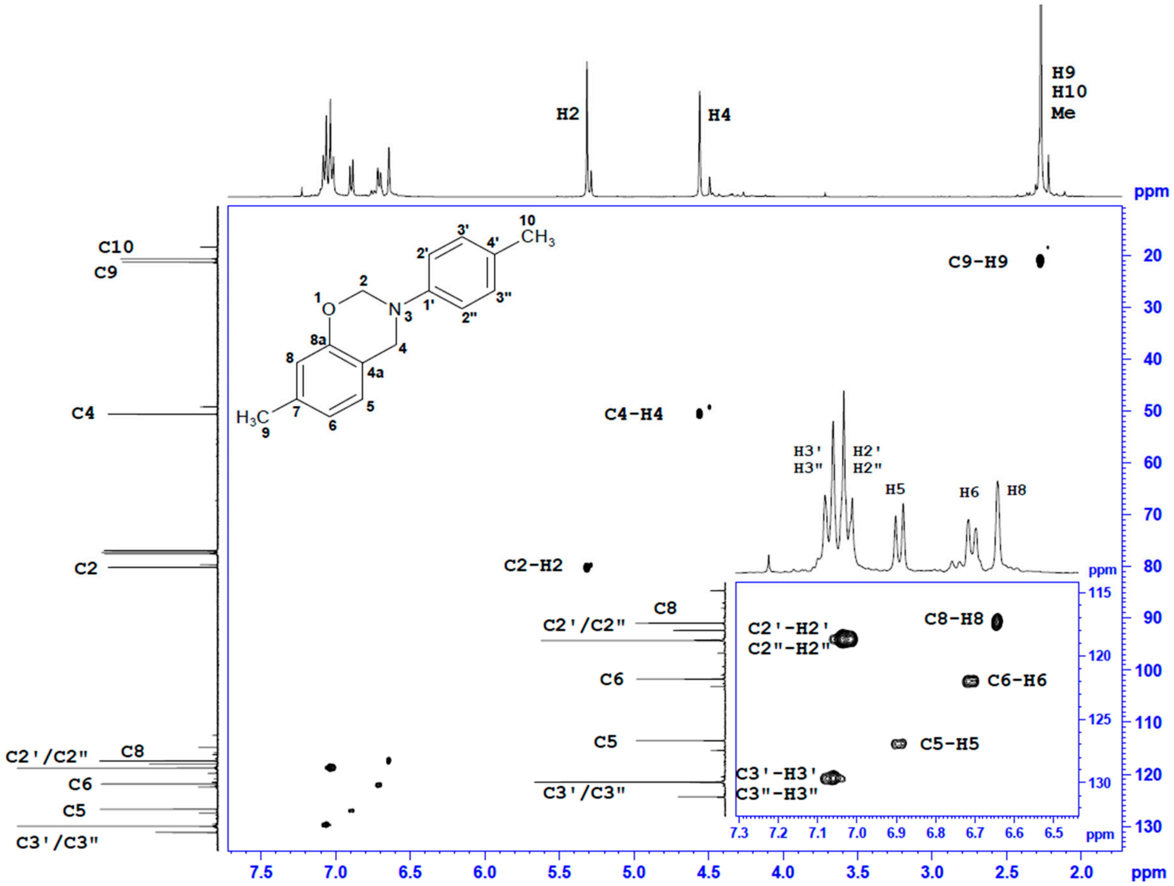

Figure S25. ^1H-^{13}C gHMBC spectrum of *m*C-*p*T benzoxazine.

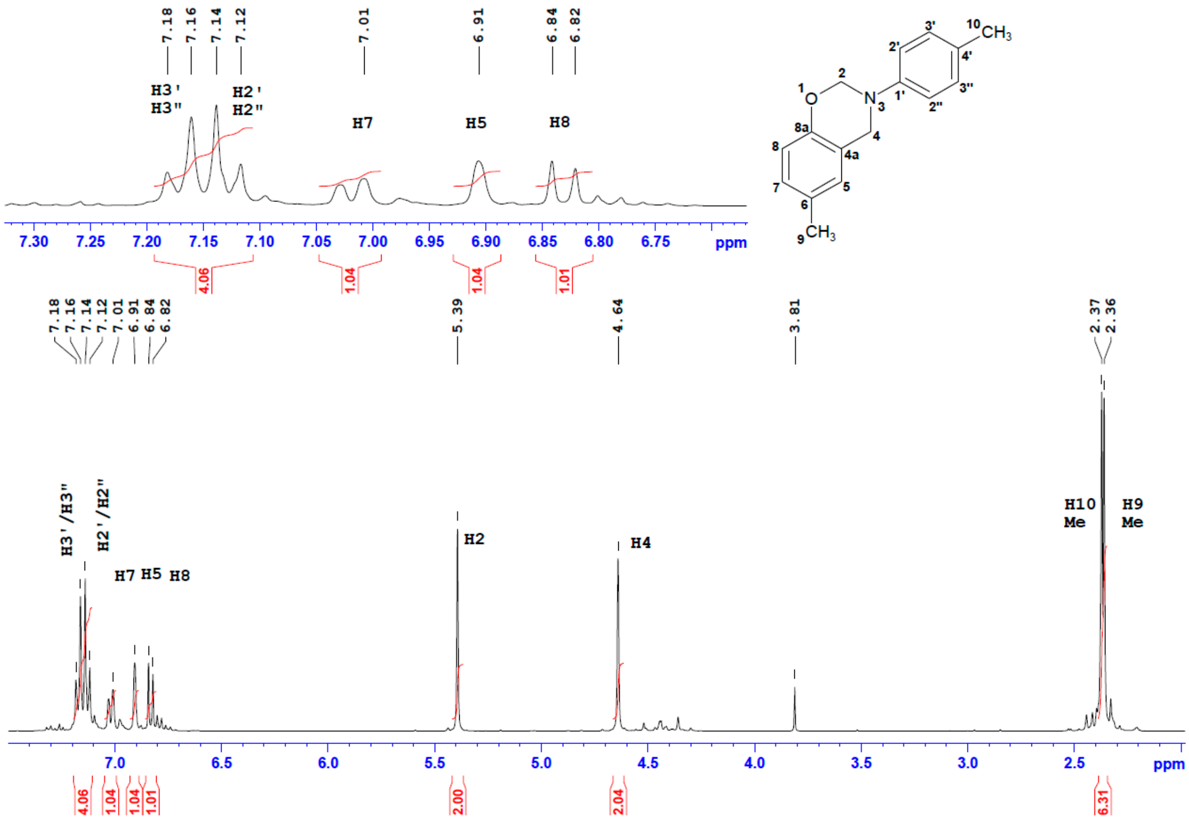

Figure S26. ^{1}H-NMR spectrum of *p*C-*p*T benzoxazine.

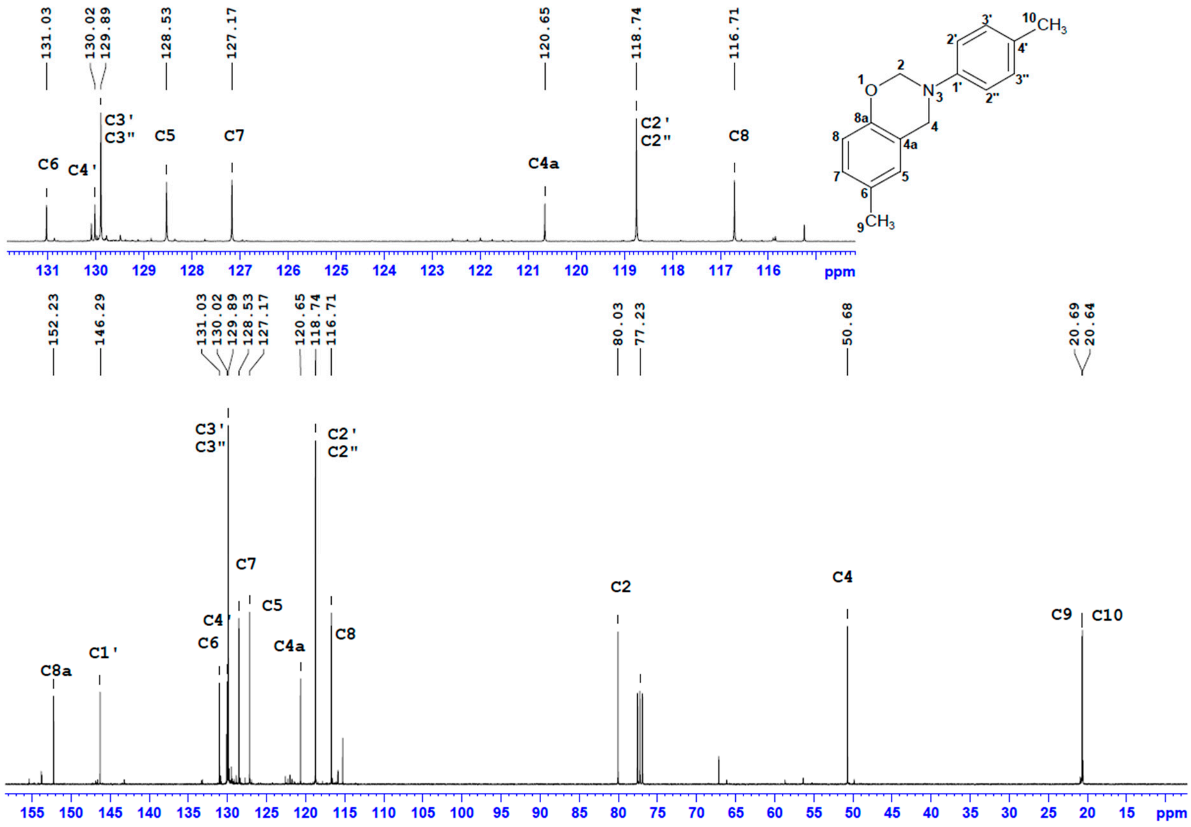

Figure S27. ^{13}C-NMR spectrum of *p*C-*p*T benzoxazine.

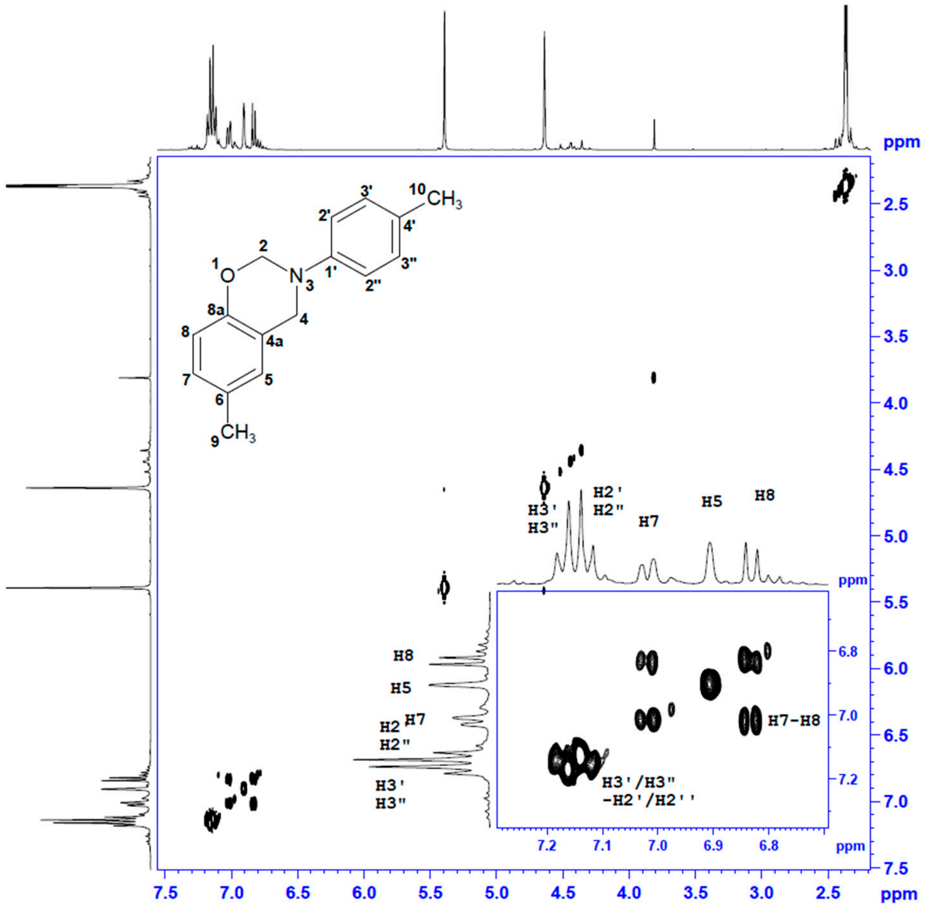

Figure S28. ^{1}H-^{1}H COSY spectrum of *p*C-*p*T benzoxazine.

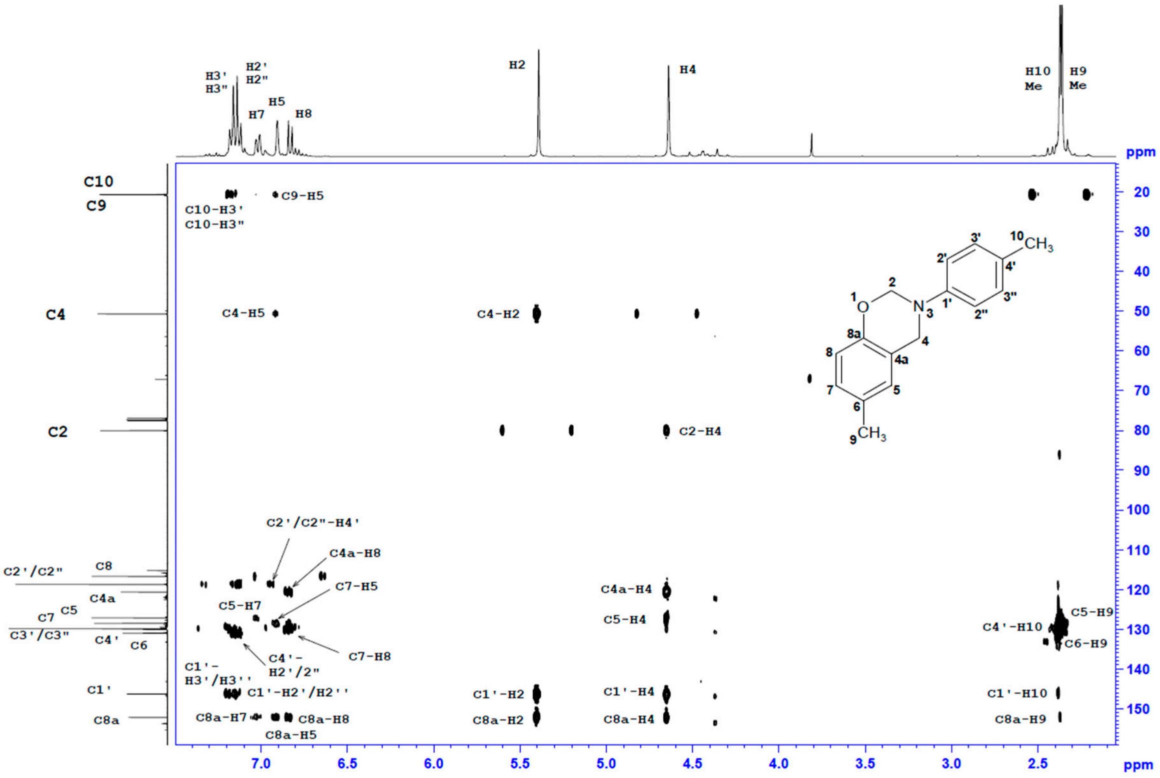

Figure S29. ^{1}H-^{13}C gHSQC spectrum of *p*C-*p*T benzoxazine.

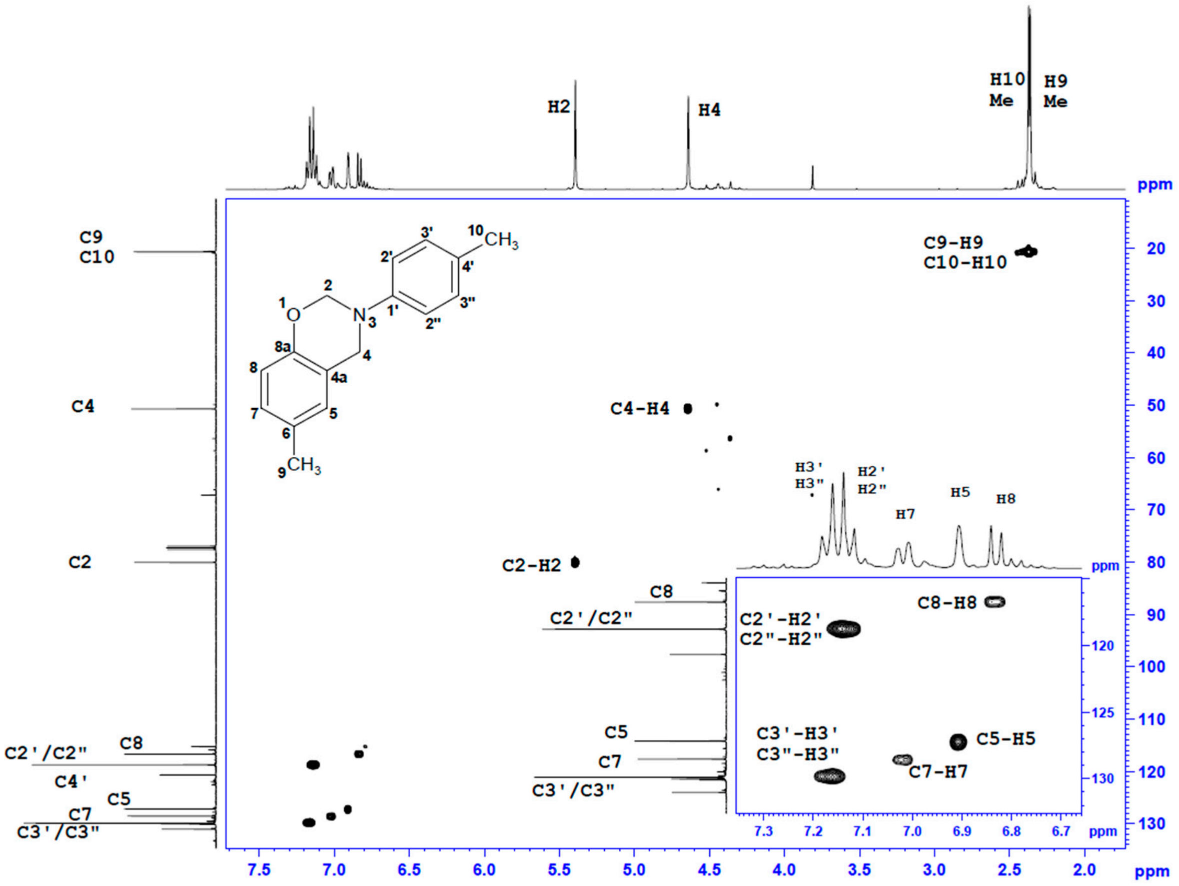

Figure S30. ^{1}H-^{13}C gHMBC spectrum of *p*C-*p*T benzoxazine.

© 2020 by the authors. Submitted for possible open access publication under the terms and conditions of the Creative Commons Attribution (CC BY) license (http://creativecommons.org/licenses/by/4.0/).
